# Measuring Fear of Falling and Appraisals of Harm

**DOI:** 10.1093/geroni/igaa057.760

**Published:** 2020-12-16

**Authors:** Helen Lach, Wanida Noimontree

**Affiliations:** 1 Saint Louis University, St. Louis, Missouri, United States; 2 Faculty of Nursing Burapha University, Mueang Chon Buri, Chon Buri, Thailand

## Abstract

Fear of falling (FOF) causes premature disability in older adults who limit their physical activities, making FOF an important area for aging research. Measures of falls self-efficacy have been widely used, but less attention has been paid to measuring the construct of fear. We re-assessed an older measure of fear and appraisals of harm to determine the factor structure and potential for a shorter version. Data was collected from 329 older adults from a variety of community setting (mean age 76.3, 84% female, 81% White); 63.8% reported FOF. Factor analysis resulted in four factors with eigenvalues greater than 1.0. Two factors achieved adequate Cronbach’s alpha: appraisals of harm outcomes (0.924) and social stigma (0.815). Further analysis was used to identify a short form, resulting in a 6 item measure of appraisal of harm outcomes (Cronbach’s alpha 0.86). The original and short versions were moderately correlated with falls self-efficacy as measured by the short form, and level of fear of falling using a 10-point rating scale (.327 - .492). Research is needed to further clarify psychological targets to help older adults remain physically active to improve quality of life.

